# Solitary plasmacytoma of mandible: an unusual bilateral presentation

**DOI:** 10.4322/acr.2021.298

**Published:** 2021-08-20

**Authors:** Sandip Kulkarni, Jashika Adil Shroff, SM Meghana, Pournima Godge, Monica Yadav, Charudatta Shridhar Naik

**Affiliations:** 1 Terna Dental College and Hospital, Department of Oral Pathology and Microbiology, Navi Mumbai, Maharashtra, India; 2 Terna Dental College and Hospital, Department of Oral and Maxillofacial Surgery, Navi Mumbai, Maharashtra, India

**Keywords:** Plasmacytoma, Plasma cells, Bilateral, Mandible, Multiple Myeloma, Case report

## Abstract

Plasmacytoma is a neoplastic proliferation of monoclonal plasma cells, which can present clinically as solitary bone neoplasm, extramedullary plasmacytoma, and multiple myeloma. The biological behavior of these tumors is variable from periods of clinical latency to rapid growth and progression from localized forms to more disseminated multiple myeloma. We present the case of solitary plasmacytoma of the mandible with rare bilateral involvement in a 65-year-old female patient. This paper highlights the importance of understanding the maxillofacial manifestations of the disease by the dentist for early diagnosis and thus better prognosis.

## INTRODUCTION

Plasma cell neoplasms are a diverse group characterized by monoclonal neoplastic proliferation of terminally differentiated B-lymphocytes.^1^^,^ 2 Plasmacytoma can present clinically as multiple myeloma (MM), solitary bone neoplasm (SBP; with single bone involvement), and extramedullary plasmacytoma (EMP; with soft tissue involvement).^3^ Solitary plasmacytoma is an isolated disease or first manifestation of a subsequent MM.^1^ SBP are uncommon and account for only 3%–10% of all plasma cell neoplasms; further involvement of jaw bones is very rare.^4^ SBP shows a three times higher male predilection (mean age 55 years).^5^ We present a rare case report of a bilateral SBP of the mandible.

## CASE REPORT

A 65-year-old female patient reported to the outpatient department of Terna Dental College, Navi Mumbai, complaining of swelling on the left side of the jaw for 1 year and on the right side over the past month. She was apparently fine 1 year ago. Her past dental history revealed extraction of teeth numbers 36 and 37 1 year ago, following which she noticed swelling in the left mandibular posterior region, which gradually enlarged to the present size. Her past medical history was noncontributory and a general physical examination revealed no abnormalities.

**Figure 1:**
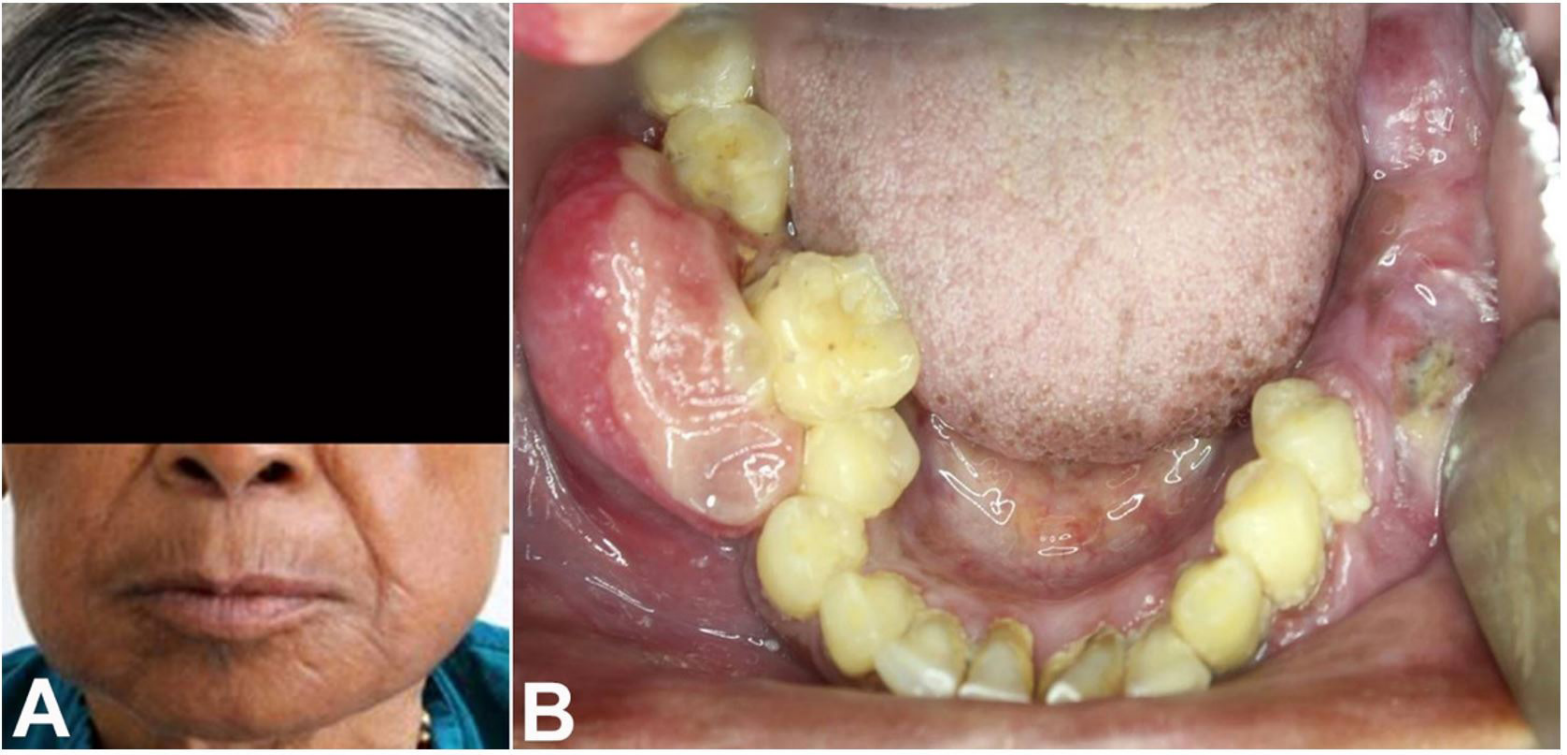
**A –** Extraoral examination showing diffuse bilateral swellings over the posterior mandible; **B –** Intraoral examination showing a proliferative mass extending from tooth number 35 to the retromolar area, and from 45 to 47.

Extra orally, diffuse bilateral swellings were noted over the lower third of the patient’s face. The overlying skin appeared to be normal. On the left side, swelling extended superior-inferiorly from 2 cm below the ala tragus line to the lower border of the mandible, and anteroposteriorly from the corner of the mouth to the anterior border of the masseter. On the right side, swelling extended from the ala tragus line to the lower border of the mandible and from the corner of the mouth to the ascending ramus (Figure1A). The swelling was firm and non-tender. The left submandibular lymph nodes were palpable, firm, and mobile.

On intraoral examination, a soft, painless, proliferating growth was seen extending posteriorly from the distal aspect of 35 to the retromolar region measuring approximately 4.5 cm × 2.5 cm. Teeth numbers 36 and 37 were missing and a soft tissue mass was found to be covering the crown of 38. Buccal vestibular obliteration with cortical expansion was observed (Figure 1B). Swelling was soft and non-tender, and showed surface ulceration because of indentations of the opposing dentition.

A right-side lesion presented as a gingival/alveolar mass on the buccal aspect of 45 to 47 measuring approximately 2 cm × 1.5 cm. The involved teeth were vital and free of any carious lesion. Growth was soft to firm in consistency with well-defined margins and an ulcerated surface.

**Figure 2:**
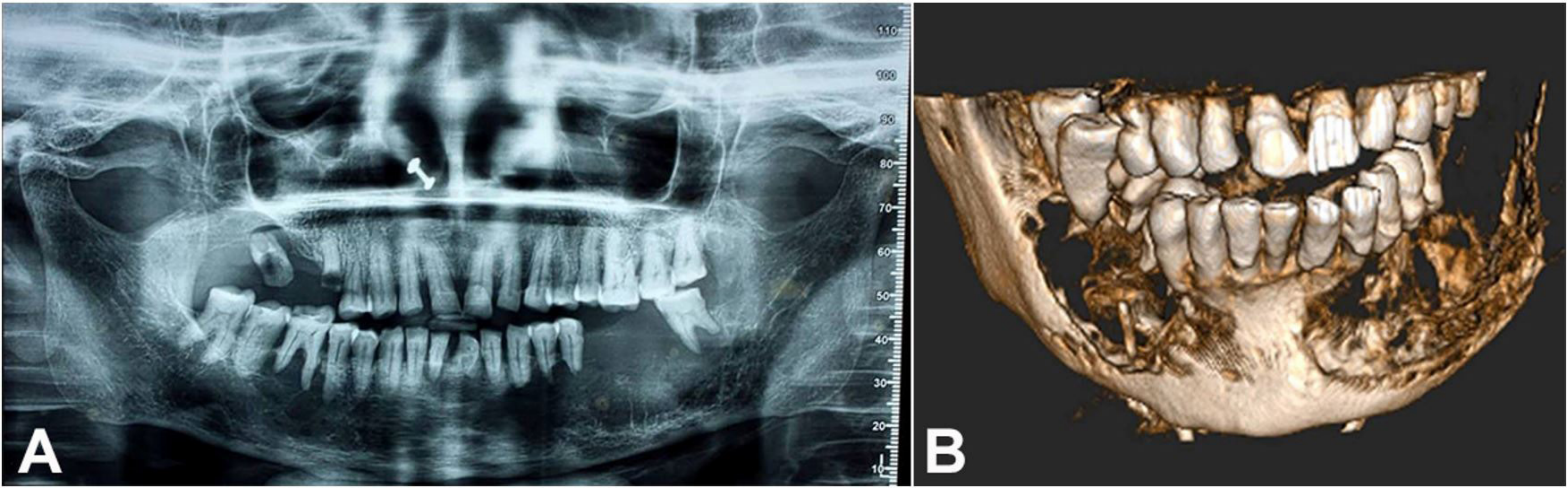
**A –** Orthopantomograph showing ill-defined radiolucency with respect to tooth numbers 31 to 38 and 45 to 48; **B –** CBCT showing the presence of bilateral expansile lytic lesions with irregular borders.

An orthopantomograph revealed the presence of bilateral ill-defined radiolucent lesions. The left-side lesion showed the involvement of the entire mandible extending from tooth number 31 to the ramus. Root resorption of involved teeth was noted. The right side showed a radiolucent lesion around the periapical area of 46 and the interdental area of 45 and 46 (Figure 2A). Cone-beam Computed Tomography (CBCT) revealed expansile lytic lesions with irregular borders. The periapical area in relation to 41 and 42 was spared (Figure 2B).

Based on the clinical and radiographic features, the provisional and differential diagnoses considered were of intraosseous carcinoma, ameloblastoma, central giant cell lesions, and lymphoma.

**Figure 3:**
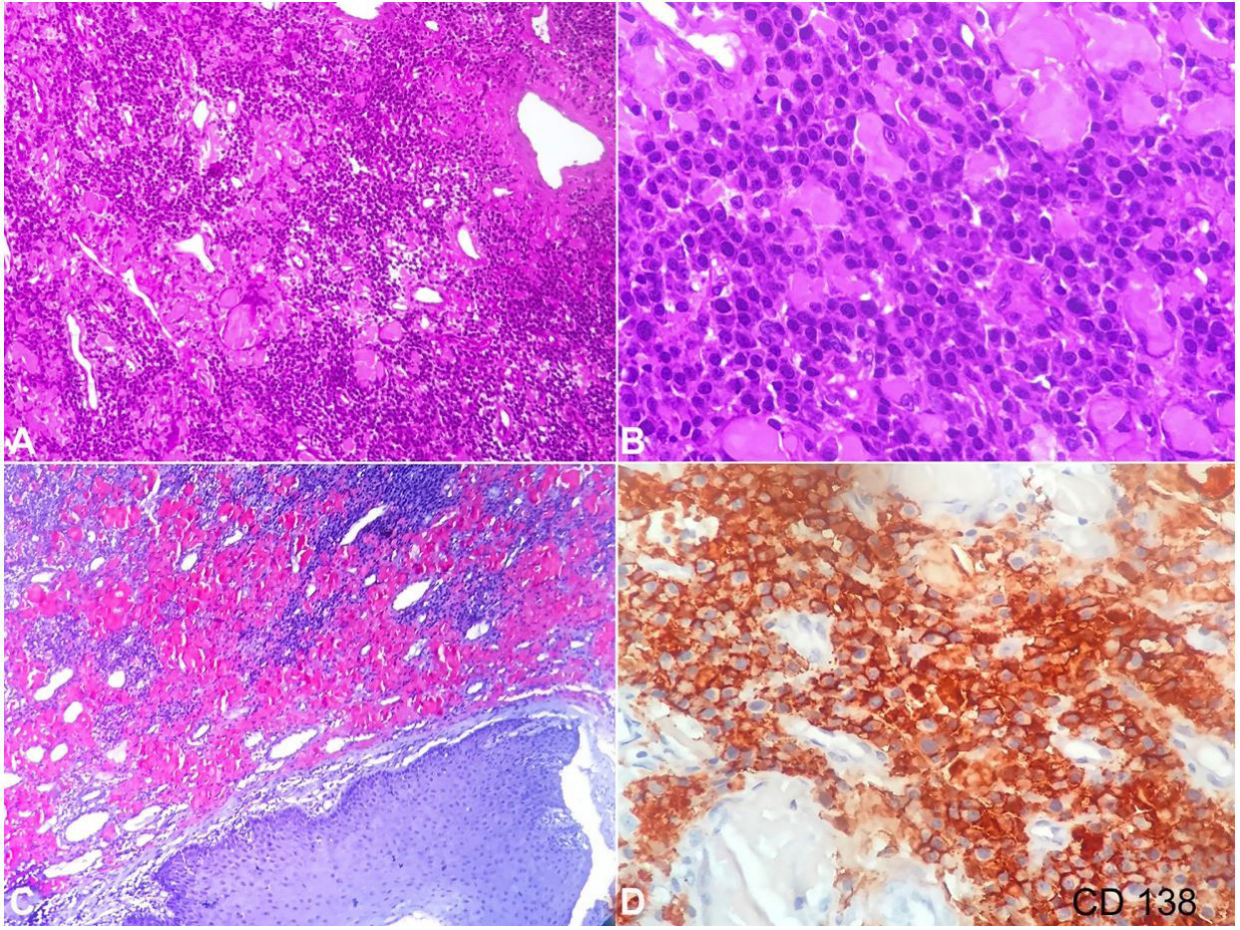
Photomicrograph of the tumor showing, **A –** Low magnification of diffuse sheets of plasma cells with eccentric nuclei (H&E x100); **B –** High magnification of atypical plasma cells with abnormal mitotic figures and occasional binucleated forms (H&E x400); **C –** amyloid deposition with in connective tissue stroma (Congo red stain x100); **D –** High Magnification immunohistochemistry showing strong positivity for CD138 (x400).

**Figure 4:**
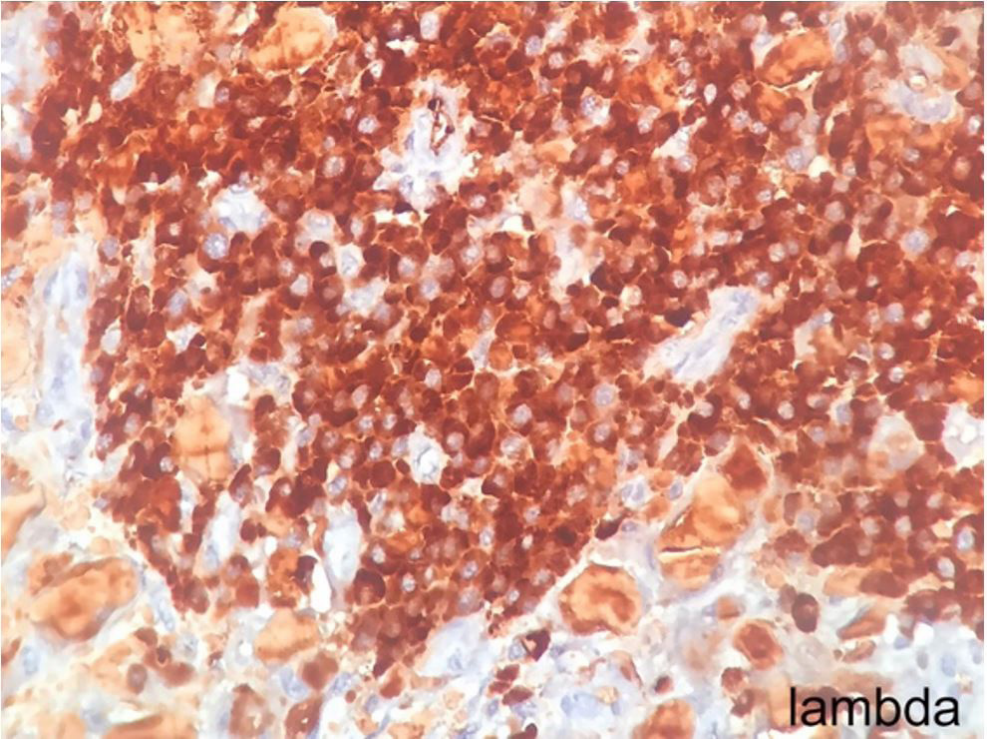
High magnification photomicrograph of the tumor showing lambda light chain positivity. (CD138 x400).

Histopathological examination of the incisional biopsy from both right and left sides showed diffuse sheets of uniformly appearing plasma cells with eccentric nuclei and pale cytoplasm (Figure 3A). Nuclear hyperchromatism and pleomorphism of cells were suggestive of a malignancy (Figure 3B). Connective tissue stroma showed interstitial amorphous eosinophilic amyloid-like material in abundance, which was later confirmed with Congo red staining (Figure 3C). Occasional giant cells were also noted. Immunohistochemistry (IHC) was performed, which revealed strong positivity for CD138 and lambda light chain; kappa chain was negative (Figures 3D and 4).

Routine hematological and biochemical investigations revealed the presence of anemia, hypocalcemia, and raised parathyroid hormone (PTH) levels. Protein electrophoresis of serum showed elevated levels of total serum protein and globulin suggestive of a characteristic M band in the gamma globulin region and hypoalbuminemia. The values of serum free light chain assay were well within normal limits. Based on these findings, the diagnosis of bilateral SBP of the mandible was made. The patient was referred to the oncology center for further systemic work-up and radiographic survey to rule out MM. Unfortunately, the patient did not survive to complete the investigations.

## DISCUSSION

Plasma cell neoplasms are lymphoid neoplastic proliferations of B cells that may be classified as MM, SBP, and EMP. SBP and EMP are a unifocal, monoclonal proliferation of plasma cells and are less aggressive compared to MM. Most SBP eventually develops into MM.^6^ In 2003, the International Myeloma Working Group published criteria for the classification of monoclonal gammopathies, MM, and related disorders recognizing SBP, EMP, and multiple solitary plasmacytomas (+/− recurrent) as distinct entities.^7^

SPB is predominantly prevalent in males with an average age of 55 years at the time of diagnosis.^5^ In contrast to the literature, the present case was reported at a slightly later age of 65 years in a female patient.

SBP primarily affects the axial skeleton. Spinal disease is observed in 34%–72% cases followed by long bones, which accounts for 20% of the cases. Jaw bones are rarely involved with more predisposition towards bone-marrow-rich areas of posterior mandible.^1^^,^ 2 ^,^ 5 ^,^ 8 The present case was reported in the posterior mandible, which is in accordance with the literature; however, bilateral involvement makes this case unique.

The most common clinical symptom of SBP is pain with paresthesia, which was not observed in our case. Swelling in hard and soft tissues, pathological fractures, hemorrhage, mobility, and migration of teeth are other manifesting features of SBP.^9^^,^ 10

The early diagnosis of SBP can be made radiographically on the basis of ill-defined destructive bone resorption attributed to the release of the osteoclastic-activating factor—a lymphokine—by clonal plasma cells.^11^ SBP can also present itself as a well-defined unilocular or multilocular lesion. Osteolytic activity results in the mobilization of calcium leading to hypercalcemia.^12^ In our case, the serum calcium level was not elevated even in the presence of extensive mandibular involvement. Anemia is a rare finding in SBP, and is probably related to hemolysis due to excess paraprotein production.^12^

Histological features of SBP and MM are identical, revealing sheets of atypical monoclonal plasma cells with various types of differentiation.^13^ Giant cells and amyloid deposition in SBP can be noticed in a few cases.^14^^,^ 15 The present case was also in accordance with previous findings. Inflammatory conditions with characteristic high plasma cell infiltrate can be differentiated from SBP by the presence of leukocytes and collagenous stroma.^16^ Poorly differentiated carcinoma and lymphoproliferative disorders may also exhibit similar microscopic features as seen in plasmacytoma. Hence the diagnosis of SBP requires confirmatory IHC analysis.^1^ IHC markers, such as LCA, EMA, and CD138, show abnormal hematopoietic activity. CD138 is a marker for plasma cells; its main reactivity in hematolymphoid neoplasms includes plasma cell neoplasms and some large B-cell lymphomas. CD45 is specific for both benign and malignant lymphoid cells.^1^ Monoclonal cytoplasmic light chain expression of malignant plasma cells differentiates it from various inflammatory conditions.^16^ In the present case, strong positivity for only lambda light chain was noted. Negative staining with CD20, CD1, NSE, S100, and HMG-45 helps to rule out epithelial, muscle, neural, histiocytic, salivary gland, and malignant melanoma.^2^^,^ 13

The current criteria to make a diagnosis of SBP are: (i) isolated areas of bone destruction due to clonal plasma cells; (ii) bone marrow plasma cell infiltration not exceeding 5% of all nucleated cells; (iii) the absence of further osteolytic bone lesions or other tissue involvement; (iv) the absence of anemia, hypercalcemia, and renal impairment; and (v) low concentrations of serum or urine monoclonal proteins.^1^^,^ 10

In our case, bilateral mandibular lesions were the only presenting features without any signs of other tissue involvement. However, the patient also presented with anemia, hypocalcemia, and immunoglobin subtype alterations, such as the presence of M band, increased total protein and globulin levels, and decreased albumin levels. Thus an early diagnosis of solitary plasmacytoma of the bone is vital for patient survival. It may represent the first manifestation of MM, as nearly 70% of cases of SBP develop into MM after initial diagnosis.^13^ Although mandibular involvement as the initial presenting sign in MM is rare, it has been reported in the literature.^11^

## CONCLUSION

SBP rarely occurs in maxillofacial areas affecting the mandible. This entity requires a meticulous overview of any signs and symptoms of systemic disease, as SBP is known to have a very high propensity to transform into MM. The importance of this lies in the appropriate sequencing of investigations—a fact that would mark a dramatic change in the treatment and prognosis of the patient.
